# Microbial and chemical dynamics of a toxic dinoflagellate bloom

**DOI:** 10.7717/peerj.9493

**Published:** 2020-07-21

**Authors:** Nastassia V. Patin, Emily Brown, Gabriella Chebli, Claire Garfield, Julia Kubanek, Frank J. Stewart

**Affiliations:** 1 School of Biological Sciences, Georgia Institute of Technology, Atlanta, GA, United States of America; 2 Center for Microbial Dynamics and Infection, Georgia Institute of Technology, Atlanta, GA, United States of America; 3 Department of Chemistry, Agnes Scott College, Decatur, GA, United States of America; 4 School of Arts and Sciences, State University of New York at Stony Brook, Stony Brook, NY, United States of America; 5 School of Chemistry and Biochemistry, Georgia Institute of Technology, Atlanta, GA, United States of America; 6 Department of Microbiology and Immunology, Montana State University, Bozeman, MT, United States of America

**Keywords:** Harmful algal bloom, Marine microbiome, Metabolomics, Microbe-phytoplankton associations

## Abstract

Harmful Algal Blooms (HABs) exert considerable ecological and economic damage and are becoming increasingly frequent worldwide. However, the biological factors underlying HABs remain uncertain. Relationships between algae and bacteria may contribute to bloom formation, strength, and duration. We investigated the microbial communities and metabolomes associated with a HAB of the toxic dinoflagellate *Karenia brevis* off the west coast of Florida in June 2018. Microbial communities and intracellular metabolite pools differed based on both bacterial lifestyle and bloom level, suggesting a complex role for blooms in reshaping microbial processes. Network analysis identified *K. brevis* as an ecological hub in the planktonic ecosystem, with significant connections to diverse microbial taxa. These included four flavobacteria and one sequence variant unidentified past the domain level, suggesting uncharacterized diversity in phytoplankton-associated microbial communities. Additionally, intracellular metabolomic analyses associated high *K. brevis* levels with higher levels of aromatic compounds and lipids. These findings reveal water column microbial and chemical characteristics with potentially important implications for understanding HAB onset and duration.

## Introduction

Oceanic bacteria and archaea interact closely with each other and with single-celled phytoplankton such as diatoms (Amin, Parker & Armbrust, 2012) and dinoflagellates (Wang et al., 2014). The latter play major roles in marine carbon and nitrogen cycles and act as agents of ecological disturbance, including through harmful algal blooms (HABs) (Field et al., 1998; Mills & Arrigo, 2010). Phytoplankton-bacterial associations include a range of mutualistic, antagonistic, and commensal interactions (Seymour et al., 2017; Cirri & Pohnert, 2019) and bacterioplankton communities change in taxonomic and functional composition with the onset of phytoplankton blooms (Pinhassi et al., 2004; Teeling et al., 2012). These interactions are driven by chemistry; for example, members of the Roseobacter clade of α-proteobacteria promote algal growth by secreting antibiotics and growth stimulants (Buchan et al., 2014; Amin et al., 2015; Johnson, Kido Soule & Kujawinski, 2016), but can shift their metabolism to the production of algicides as blooms progress (Seyedsayamdost et al., 2011; Segev et al., 2016). Despite this emerging picture of dynamic algal–bacterial chemical associations, their role in the progression of phytoplankton blooms is not well understood (Seymour et al., 2017; Cirri & Pohnert, 2019).

HABs can cause significant shifts in microbial community structure and interactions. These transformations involve diverse chemical processes, including both positive and negative interactions between microbes and algae. For example, blooming algae convert CO_2_ and inorganic nutrients, such as nitrogen and phosphorus, into organic matter, much of which is directly processed by bacteria (Buchan et al., 2014). A shift in coastal waters from oligotrophic to nutrient- and organic-rich leads to increased heterotrophic microbial activity as well as successive shifts in microbiome composition and metabolism corresponding to various stages of algal-derived organic matter decomposition (Teeling et al., 2012; McCarren et al., 2010). Conversely, bacteria can stimulate phytoplankton growth by the remineralization of organic matter (Cho & Azam, 1988; Burkhardt et al., 2014), as well as directly inhibit growth through the production of algicidal compounds (Mayali & Azam, 2004; Aiyar et al., 2017; Meyer et al., 2017). Microbes can provide micronutrients that phytoplankton are unable to synthesize, including B vitamins (Tang, Koch & Gobler, 2010; Kazamia et al., 2016) and siderophores that provide biologically available iron to both the microbial producers and associated algal cells (Boiteau et al., 2016; Basu et al., 2019). Phytoplankton themselves may produce compounds that mediate ‘positive’ interactions. For example, polyunsaturated aldehydes from diatoms stimulate certain metabolic activities of heterotrophic bacteria, including respiration and organic matter hydrolysis, thereby impacting carbon export efficiency (Edwards, Bidle & Mooy, 2015). Additional co-dependent interactions may be common in marine microbial communities, but likely have escaped attention in the absence of studies that test for co-variation among community members and metabolite profiles.

Blooms of the toxic dinoflagellate *Karenia brevis* are an important model for assessing changes in phytoplankton-associated microbial communities and metabolites. *K. brevis* synthesizes the polyether brevetoxins, potent neurotoxins that cause mass mortality of fish, marine mammals, sea turtles, and birds (Landsberg, 2002). During blooms, *K. brevis* cell density can reach 5 × 10^6^ cells L^−1^ with correspondingly high brevetoxin levels (Walsh et al., 2006). Like many phytoplankton, *K. brevis* has been shown to associate with bacteria and grow poorly in their absence (Roth, Mikulski & Doucette, 2008; Roth et al., 2008). Furthermore, *K. brevis* produces allelopathic compounds that negatively affect the metabolism of competitor algae (Prince, Myers & Kubanek, 2008; Poulson-Ellestad et al., 2014). However, the effects of these compounds on associated bacteria and the strength of *K. brevis*-microbe interactions in the environment remain unclear.

Recent advances in computational methods for analyzing ‘-omics’ data offer an unprecedented opportunity to study the linkages between biological and chemical features of algal blooms. Both molecular and metabolomic data are highly complex and traditional ecological statistics are often unsuited to analyzing diversity patterns (Thorsen et al., 2016; Tsilimigras & Fodor, 2016; Gloor et al., 2017; Weiss et al., 2017). However, improvements in data science, including statistical methods that account for the noisy and sparse character of high-throughput sequencing data sets, have spurred interest in comparative microbiome studies (Love, Huber & Anders, 2014; McMurdie & Holmes, 2014; Kyrpides, Eloe-Fadrosh & Ivanova, 2016; Amir et al., 2017; Bolyen et al., 2019). Furthermore, linking metabolomes with microbiomes provides deeper insight into questions of ecological function. Combining community molecular and metabolomic data has improved our understanding of spatial and temporal biological and chemical distributions on the human skin (Bouslimani et al., 2015), in a diseased lung (Garg et al., 2017), and among objects and surfaces of human habitats (Kapono et al., 2018). In the surface ocean, events such as phytoplankton blooms provide important opportunities for addressing questions of microbe-chemical distributions and associations.

Here, we investigated the relationships between phytoplankton and bacterial communities and their intracellular metabolism during one of the most severe and long-lasting *K. brevis* blooms in recent history. This bloom took place off the Gulf coast of Florida, a region regularly impacted by *K. brevis* HAB events. It began in November 2017 and persisted, with varying degrees of intensity, until January 2019 (Weisberg et al., 2019). Ecological impacts from the bloom were severe and included mass mortalities of fish, birds, and other wildlife. Our sampling targeted a period of bloom resurgence in June 2018 and spanned sites of varying *K. brevis* population density (bloom level) around the mouth of Tampa Bay. Our overarching goals were to assess the extent to which community metabolomes and microbiomes vary with bloom level and to identify ecologically relevant microbe-algae associations.

## Experimental Methods

### Sampling sites, filtration, and preservation

**Figure 1:**
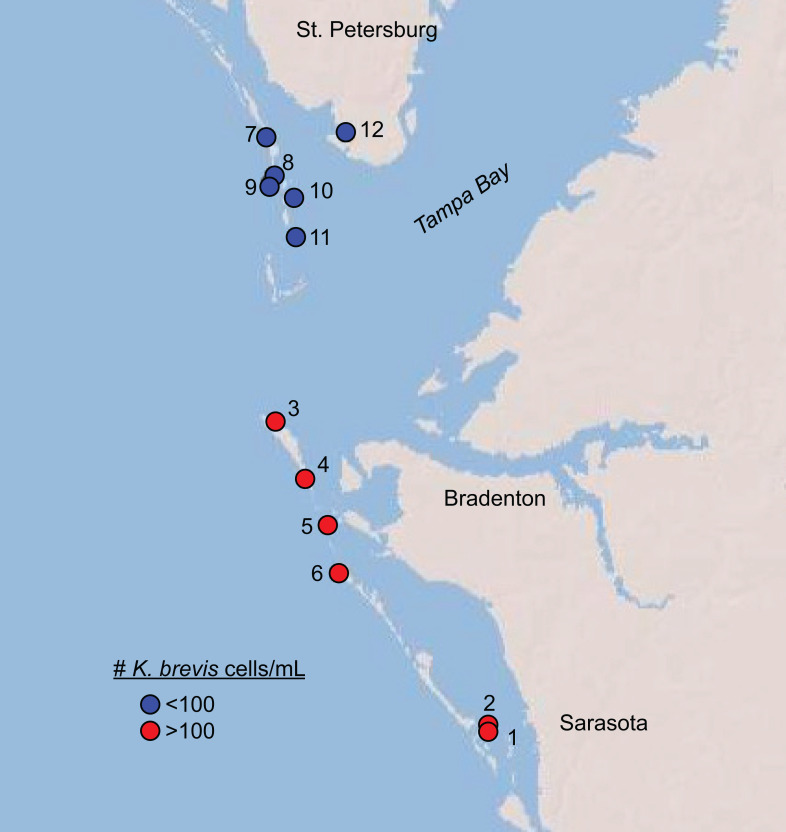
Map of sites sampled in this study with sites, colored by *K. brevis* level. Map of sites sampled in this study. All sites along Longboat Key (Sites 1–6) featured >100 *K. brevis* cells/mL and all sites north of the entrance to Tampa Bay (Sites 7–12) featured 0–100 *K. brevis* cells/mL.

Over two days in June 2018, we sampled twelve shoreline sites near the mouth of Tampa Bay, Florida for phytoplankton counts, microbial DNA, and chemical metabolites (Fig. 1, Table S1). This time period marked the beginning of a new surge in *K. brevis* abundances after 8–10 weeks of low (<100 cells/mL) cell counts along Sarasota and Manatee County coastlines (Fig. S1), although the bloom was technically still in progress statewide both before and after our sampling. Six sites were north of Tampa Bay; six were south of Tampa Bay, and in or around Sarasota Bay. The sites represented two levels of a *Karenia brevis* bloom based on Florida Fish and Wildlife Conservation Commission (FWC) reports at the time of sampling (https://myfwc.com/research/redtide/statewide/). Surface currents and salinity distributions were obtained from the Finite Volume Community Ocean Models made available by the Ocean Circulation Group at the University of South Florida (http://ocgweb.marine.usf.edu/Models/FVCOM/month_tb_ch.php?year1=2018&mon1=jun).

Sites included docks within sheltered bays, ocean-facing beaches with high wave action, and semi-sheltered sites near bay entrances (Table S1). At each site, approximately 3 L of surface water was collected by hand from immediately offshore, either by wading from the beach into the surf zone (1–2 m depth) or from a dock. Water was stored in autoclaved polystyrene screw-top bottles at room temperature in the dark for 1–5 h before transportation to Mote Marine Laboratory for processing. Additionally, at each site two replicate samples of 20 mL unfiltered water were preserved in glass scintillation vials with 200 µL acidic Lugol’s solution (5 g I_2_, 10 g KI, 100 mL DI water, 10 mL glacial acetic acid; final iodine concentration 1%) for microscopy and stored in the dark at room temperature for 2–4 days until processing at Georgia Tech.

Each 3 L sample was pre-filtered through a 30 µm pore size filter to remove any detritus or larger organisms. From this filtrate, 200 mL was processed for metabolomic analysis by sequential filtration onto a 3.0 µm nitrocellulose filter (MilliporeSigma) and a 0.2 µm polycarbonate filter (MilliporeSigma). Both filters were 47 mm in diameter and were stored in glass scintillation vials with methanol (enough to submerge filter) at room temperature until transport to Georgia Tech 1–2 days after sampling, where they were then stored at 4 °C until processing four weeks later.

The remaining ∼2800 mL of filtrate was processed for molecular analysis by sequential filtration onto 3.0 µm nitrocellulose (MilliporeSigma) and 0.22 µm Sterivex (MilliporeSigma) filters. The 3.0 um filters were 47 mm in diameter and housed in Swinnex (MilliporeSigma) filter holders. Filters were preserved with approximately three mL of DNA/RNA stabilization buffer (25 mM sodium citrate, 10 mM EDTA, 5.3 M ammonium sulfate (pH 5.2); (Patin et al., 2018) in five mL CryoTubes (ThermoFisher) and stored at −80 ° C or on dry ice until further processing at Georgia Tech.

Storage and handling time were minimized as much as possible while balancing the importance of treating all samples identically. It is possible that the samples underwent biological or chemical changes (bottle effects) during the time (1–5 h) between water collection and filtration. This is a common trade-off faced by researchers in field studies (Scofield et al., 2015). Bottle effects, if they occurred, would most likely result in a type II (false negative) error, assuming they operated relatively uniformly across samples and were larger than differences due to bloom state.

### Phytoplankton cell counts

From each 20 mL sample preserved with Lugol’s solution, the settled cells were enumerated using a 10 mL settling chamber (Hydro-Bios). Cells were settled overnight before counting with an Olympus IX-50 inverted microscope (400X magnification). Phytoplankton cells were identified and quantified under the following categories: diatom single cells, diatom colonies, *K. brevis*, and other (non-*K.brevis*) dinoflagellates.

### DNA extraction, PCR amplification, and sequencing

DNA was extracted from the filters according to the protocol in (Patin et al., 2018). Briefly, the preservation buffer was flushed from the Sterivex cartridges and replaced with two mL DNA lysis buffer and 40 µL of lysozyme. The membranes (3 and 30 µm pore size) were similarly rinsed and combined with five mL lysis buffer and lysozyme in 15-mL Falcon tubes. Filters were incubated and rotated at 37 °C for 45 min, after which 100 µL of proteinase K and 100 µL of SDS (20%) were added, followed by a 2-hr incubation and rotation at 55 °C. Sterivex cartridges were then flushed with approximately three mL of lysis buffer, and the combined elution volume was transferred to 15 mL centrifuge tubes. Membrane filters were removed from the Falcon tubes, and the remaining solution was used for extraction. Three mL of phenol:chloroform:isoamyl alcohol (25:24:1) were added before vortexing and centrifugation for 5 min at 3500xG. Following centrifugation, the aqueous layer was transferred to a new 15 mL centrifuge tube. Three mL of chloroform:isoamyl alcohol were added and the samples were vortexed. The samples were centrifuged at 2500xG for 5 min. For each sample, the aqueous layer was transferred to an Amicon Ultra-4 centrifuge filter (10 kDa pore size). These were centrifuged at 3500xG until the volume was under 250 µL. TE buffer ( four mL) was added as a rinse and the samples were again centrifuged at 3500xG until the final volume was below 100 µL.

Purified DNA was quantified on a Qubit fluorometer (Life Technologies) and all samples were standardized to a concentration of 4 ng/µL. Sequencing libraries were generated by PCR amplification of the V4 region of the 16S rRNA gene using Earth Microbiome Project primers 515F (Parada, Needham & Fuhrman, 2016) and 806R (Apprill et al., 2015) in a protocol adopted from Kozich et al. (2013). Each PCR contained 21 µL Platinum Taq HiFi Mix (Invitrogen), 1 µL BSA, 0.5 µL each of the forward and reverse primers (10 µM), and 2 µL template DNA. Thermal cycling consisted of initial denaturation for 1 min at 94 °C, followed by 24 cycles of 30 s at 94 °C, 30 s at 55  °C, and 60 s at 68 °C, and a final step of 60 s at 68 °C. Due to poor amplification, DNA from the 30 µm filter samples from sites 2, 3, 4, and 6 was subjected to 30 PCR cycles using a modified protocol involving a 45 s annealing step at 55 °C and a 90 s extension step at 68 °C. Successful amplification was confirmed by agarose gel electrophoresis. We failed to amplify DNA from the largest (>30 µm) size fraction from sites 2, 3, and 4. Amplicons from all other samples were combined in equimolar concentrations, and the combined library was purified using AMPure XP beads (Beckman Coulter) and sequenced on an Illumina MiSeq using a v2 500 cycle kit and a 2 x 250 bp paired-end protocol according to (Patin et al., 2019) and the manufacturer’s instructions.

### Bioinformatic processing

Amplicon sequences were quality-filtered using Trimmomatic v. 0.36 (Bolger, Lohse & Usadel, 2014) as in (Patin et al., 2019) with the following parameters: removal of bases at the start of a read below quality 3 (LEADING:3), removal of bases at the end of a read below quality 3 (TRAILING:3), read scanning with a 4-base sliding window cutting when average quality per base drops below 25 (SLIDINGWINDOW:4:25), and minimum read length of 150 bp (MINLEN:150). The intermediate size fraction (3–30 µm) dataset from site 12 contained only 92 sequences after quality filtering and was excluded from further analyses (Table S1). Quality filtered sequences were analyzed with the QIIME2 pipeline (qiime2.org) (Bolyen et al., 2019) by applying Deblur algorithm (Amir et al., 2017), which uses sequencing error profiles to remove likely artifacts instead of clustering them in operational taxonomic units. The resulting individual sequence variants (SVs) represent distinct taxa. SV taxonomy was assigned using a Bayes classifier trained on the “Silva 132 99% OTUs from the 515FB/806RB region” database (QIIME2 2018.6 version), and sequences classified as chloroplasts were removed, along with sequences that could not be assigned to a domain. A phylogeny was generated with FastTree 2 and used for calculating phylogeny-based diversity metrics, including weighted and unweighted Unifrac (Lozupone et al., 2011).

To estimate eukaryotic phytoplankton composition, we further analyzed the fraction of the QIIME2-processed sequences that were classified as chloroplast 16S rRNA. For these sequences, we first extracted the corresponding full length amplicons (post-quality filtering but pre-Deblur processing). We then compared these longer sequences to the PhytoRef database (phytoref.sb-roscoff.fr) (Decelle et al., 2015) using BLAST v. 2.2.28+ (Camacho et al., 2009) with max_target_seqs set to five. Taxonomic identity was assigned based on top BLAST match (highest % sequence similarity). For queries having multiple top matches (taxa) with identical % similarity, taxonomy was assigned based on the taxon most likely to occur in Gulf coastal waters (based on literature). In three cases, a query had two top matches, both of which were equally likely to occur at the sampling site; for these, we noted the ambiguity by retaining both taxonomic names.

### Statistical analyses

Alpha diversity (Shannon diversity and Pielou’s evenness) was calculated in QIIME2 based on 10,000 reads per sample. Diversity values were compared between size fractions and bloom levels using box plots generated in R using the ggplot2 package (Wickham, 2016).

SV counts were transformed using the varianceStabilizingTransformation function in the DESeq2 library (Love, Huber & Anders, 2014) in R to obtain a matrix of counts that were approximately homoscedastic. A principal components analysis (PCA) was performed with the resulting matrix based on singular value decomposition using the prcomp function in R. Points on the PCA plot were colored according to bloom level and size fraction. A two-way permutational analysis of variance (PERMANOVA) was performed to test for significant effects of both size fraction and bloom level using the vegan package in R (Oksanen et al., 2019).

To obtain an overview of taxonomic trends among size fractions and between high and low bloom sites, box plots were generated using the Seaborn package in iPython. The small size fraction dataset from site 12 was excluded from these comparisons because it featured several major taxa with outlier trends compared to all other sites (the intermediate sample from this site was already excluded due to low sequence counts). We included the small size fraction from site 12 in all statistical analyses described elsewhere.

To statistically test for SVs differentially abundant between the two bloom conditions, raw SV counts were used to generate a beta-binomial regression model with the corncob package in R (Martin, Witten & Willis, 2019). The beta-binomial model is uniquely appropriate for amplicon sequence data as it allows for zero values (absences of an SV in samples), variability in per-sample total counts, and overdispersion in relative abundance values (a common property of microbiome data). Furthermore, the model allows for detection of both differentially-abundant and differentially-variable taxa. Here, both were identified with the differentialTest function using the Wald likelihood ratio test with no parametric bootstrap algorithm and a desired Type I error rate (fdr_cutoff) of 0.05. Separate analyses were run for the small and intermediate size fractions.

To test for significant associations between taxa, correlation networks were generated in the program Maximal Information Nonparametric Exploration (MINE) (Reshef et al., 2011). MINE identifies a range of correlation function types (e.g., linear, exponential, or periodic) based on maximal information coefficient (MIC) values calculated from raw SV counts. *K. brevis* counts (based on microscopy) were included in this analysis along with the SV count data. Separate analyses were run for the small and intermediate size fractions. For each, all pairwise association values above the statistically significant level (as determined by sample size-based *p*-values) were extracted from the results. Of these, associations with an MIC value greater than 0.85 were used to generate networks in Gephi (Bastian, Heymann & Jacomy, 2009). A cutoff of 0.85 was chosen because it limited networks to visually interpretable sizes. Undirected networks were generated using a Fruchterman Reingold layout and modularity was calculated using a randomized community detection algorithm with a resolution of 1.0. Nodes colored by modularity class and sized by average weighted degree. All nodes connected to *K. brevis* were labeled with font size proportional to the weighted degree of the node. For the intermediate size fraction, the nodes of five modules with no connections to the main network were also labeled.

### Extract processing for metabolomics

The 0.2 and 3.0 µm disk filters were stored at 4  °C upon arrival at Georgia Tech until extraction using a protocol modified from Poulson-Ellestad et al. (2014). Filters were flash frozen with liquid nitrogen and ground with a mortar and pestle, and metabolites were extracted with methanol (LC/MS grade). Filter particulates were pelleted by centrifugation and the methanol extract was transferred to a fresh vial. The filter pellet was then rinsed 3 times with methanol (LC/MS grade). Methanol extracts were filtered through 0.2 µm polytetrafluoroethylene (PTFE) disk filters to remove any remaining suspended particles and then dried *in vacuo* on a ThermoSavant SpeedVac vacuum concentrator. Dried methanol extracts were stored at −20 °C until next steps. The dried extracts were then re-dissolved in a chloroform/water/methanol solution and partitioned twice more with water and methanol, resulting in a 19:27:30 solvent ratio. The chloroform/methanol and methanol/water phases were each filtered through a 0.2 µm nylon disk filter and dried on a ThermoSavant SpeedVac vacuum concentrator, resulting in a lipid-soluble extract (from the chloroform/methanol phase) and an aqueous extract (from the methanol/water phase).

### ^1^H NMR spectroscopy and metabolomics analysis

^1^H NMR spectral data were acquired from extracts representing a uniform concentration of ∼3 X 10^5^ *K. brevis* cells/mL based on microscopy counts (Table 1). The lipid-soluble extracts were prepared for analysis in deuterated chloroform containing 1% trimethylsilane (TMS); the aqueous extracts were too dilute for NMR spectroscopy. ^1^H NMR spectra of lipid-soluble extracts were acquired on an 800 MHz Bruker Ascend™ NMR spectrometer using 400 scans per sample with a spectral width of 12 ppm centered at 5.5 ppm. Spectra were aligned by setting the internal standard peak (TMS) at 0.0 ppm, manually phased, baseline corrected, and segmentally aligned. For both the small (0.2–3.0 µm) and intermediate (3.0–30 µm) size fractions, spectral regions around TMS (−0.3 to 0.3 ppm), a negative peak (5.45–5.50 ppm), the residual chloroform signal (7.235–7.285 ppm for the small size fraction, 7.20–7.325 ppm for the intermediate fraction), and the excess area at the end with no peaks (8.0–11.5 ppm) were excluded from analysis. Spectral regions around containments (7.65 to 8.00 ppm) were also excluded from the small size fraction. Before multivariate analysis, NMR spectra were binned (0.005 ppm) and preprocessed via probabilistic quotient normalization, generalized logarithmic transformation, and mean-centering. Orthogonal partial least squares discriminant analysis (PLS-DA) was used to investigate variance in the chemical profiles between lipid-soluble extracts of samples classified as “high” (>100 *K. brevis* cells/mL) and “low” (<100 *K. brevis* cells/mL) bloom (MATLAB, version 8.1.0, PLS Toolbox, version 8.0.2; Eigenvector Research).

**Table 1:** Phytoplankton cell counts at each collection site show a range of *K. brevis* abundances.

	Cells/mL			
**Site**	***K. brevis***	**Other dinoflagellates**	**Diatoms(solitary)**	**Diatoms(colony)**
1	516	0	727	1001
2	357	15	349	451
3	1463	7	102	36
4	182	0	73	0
5	524	0	735	960
6	109	0	262	58
7	7	0	589	211
8	36	0	284	44
9	65	0	211	138
10	22	0	1200	975
11	36	0	421	415
12	0	11,400	626	691

### Sparse canonical correlation analysis of microbiome and metabolome data

To identify features driving the composition of both microbiome and metabolome data, transformed SV counts and NMR peak intensities were combined in a sparse canonical correlation analysis (sCCA) using the PMA package in R (Witten & Tibshirani, 2009). This method compares sets of features across high-dimensional data tables and identifies a subset of features that capture the most covariance (i.e., signals present in both tables) (Holmes & Huber, 2019). Samples (microbiome or metabolome) with counts of zero for six or more features were filtered out of the matrices and the sCCA function was run with the following sparsity penalties: small size fraction penaltyx = 0.2 and penaltyz = 0.05, intermediate size fraction penaltyx = 0.1 and penaltyz = 0.15. Separate analyses were run for the small and intermediate size fractions.

## Results

### Phytoplankton community structure

*Karenia brevis* population densities ranged from 0 to 1463 cells/mL among sites sampled over two days in June 2018 (Fig. 1, Table 1, Table S1). All sites south of Tampa Bay (#1–6) had levels above 100 cells/mL, corresponding to a “medium” bloom level designated by the Florida Fish and Wildlife Conservation Commission (FWC). All sites north of the entrance to Tampa Bay (#7-12) contained fewer than 100 cells/mL, with site 12 being the only site with no detectable *K. brevis* cells. We therefore divided the sample sites into two groups: those associated with “high” or “low” *K. brevis* bloom levels, corresponding to southern versus northern sites, respectively (Fig. 1). Oceanographic models showed a relatively modest, but uniform, influence from lower salinity bay waters. All sites lay between the same salinity isolines at the time of sampling (33-34 psu), with the exception of site 12, which was situated between 31 and 32 psu isolines during the sampling time (http://ocgweb.marine.usf.edu/Models/FVCOM/month_tb_ch.php?year1=2018&mon1=jun).

Other (non-*K. brevis*) dinoflagellates were largely absent from samples, with the exception of the site 12 sample, which contained >11,000 cells/mL belonging to the genus *Gonyaulax* while lacking *K. brevis* altogether (Table 1). Unicellular diatom abundances ranged from 73 to 1,200 cells/mL, and colonial diatom abundances ranged from 0 to 1001 cells/mL (Table 1). The abundances of phytoplankton groups as categorized here were not correlated with *K. brevis* levels (correlation values not shown).

Taxonomic analysis of chloroplast 16S rRNA gene sequences confirmed the presence of several groups of diatoms in all samples, including the marine families Chaetoceraceae, Cymatosiraceae, and Triceratiaceae (Table S2). Other phytoplankton identified by chloroplast sequences included chrysophytes, also called golden algae, and prymnesiophytes, which include coccolithophores. No *Karenia brevis* or *Gonyaulux* sp. chloroplast sequences were identified.

### Microbiome diversity, composition, and connectivity

Sequence read counts following trimming and quality filtering ranged from 10,961 to 66,447 per sample (excluding the site 12 sample from the 3–30 µm size fraction, which was removed due to low counts) (Table S1). Post-Deblur counts ranged from 4,348 to 33,394 (Table S1).

**Figure 2:**
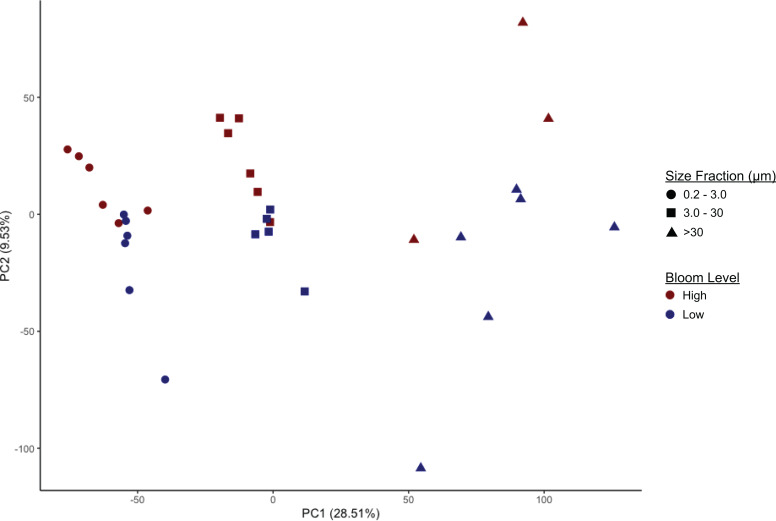
Separation of microbiomes by size fraction and bloom level by two principal components. Microbiomes separate along the first principal component (PC1, x-axis) by size fraction (0.2–3 µm, 3–20 µm, or >30 µm) and along the second principal component (PC2, y-axis) by *K. brevis* bloom level (“High” ≥100 cells/mL, “Low ≤100 cells/mL).

Microbiome community composition and alpha diversity were strongly influenced by size fraction (0.2–3 µm, 3–30 µm, and >30 µm) (Fig. 2, Figs. S2, S3; PERMANOVA *p* = 0.001). Measures of Faith’s phylogenetic diversity were highest in the largest fraction and lowest in the free-living (0.2–3.0 µm) fraction. Similarly, in most cases, taxonomic groups in the intermediate size fraction (3–3.0 µm) were at intermediate relative abundance compared to abundance in the smallest and largest size fractions; i.e., there were only a few taxonomic groups enriched or depleted in the intermediate fraction compared to the other two fractions. These included Euryarchaeota (Marine Group II clade, hereafter referred to as MGII) and, most notably, the class Cyanobacteria (phylum Cyanobacteria, hereafter referred to as cyanobacteria), both of which were present in higher proportions in the intermediate fraction (Figs. S2, S4). In comparison to the particle-associated (3–3.0 and >30 µm) fractions, the free-living fraction contained relatively more Proteobacteria (of all classes, excluding Deltaproteobacteria, which was most abundant in the largest size fraction), Deferribacteres, and Acidobacteria, but relatively fewer cyanobacteria and members of the phylum Bacteroidetes, particularly of the class Sphingobacteria (Figs. S2, S4). The largest (>30 µm) size fraction contained higher proportions of Verrucomicrobia and Planctomycetes, notably Planctomycetes of the uncultured class OM190 (Fig. S4), but lower proportions of Actinobacteria and Archaea compared to the other fractions. The >30 µm fraction also contained a substantial portion (2–10%) of a taxon unclassified beyond the domain Bacteria (Fig. S4).

Community composition also differed significantly based on bloom level (>100 cells/mL or “high”, vs. <100 cells/mL or “low”; PERMANOVA *p* = 0.002). This effect was observed within each size fraction, although separation by bloom level was less robust compared to separation by size fraction, with some overlap among the high and low bloom level microbiomes (Fig. 2, Table S3). Alpha diversity, however, did not differ significantly between bloom levels (Fig. S3, Table S4).

Several broad taxonomic groups differed notably in relative abundance between high and low bloom conditions (Fig. S5), with these differences most pronounced in the particle-associated fractions. In the free-living size fraction, taxon-specific variation based on bloom level was minor and evident only for the classes Flavobacteriia (phylum Bacteroidetes, hereafter referred to as flavobacteria) and Acidobacteria (Fig. S5). In the intermediate size fraction, flavobacteria, Deferribacteres (phylum Deferribacteres), and Deltaproteobacteria were differentially abundant between high and low bloom sites (Fig. S5). Of these taxa, only flavobacteria were enriched in high bloom sites.

**Figure 3:**
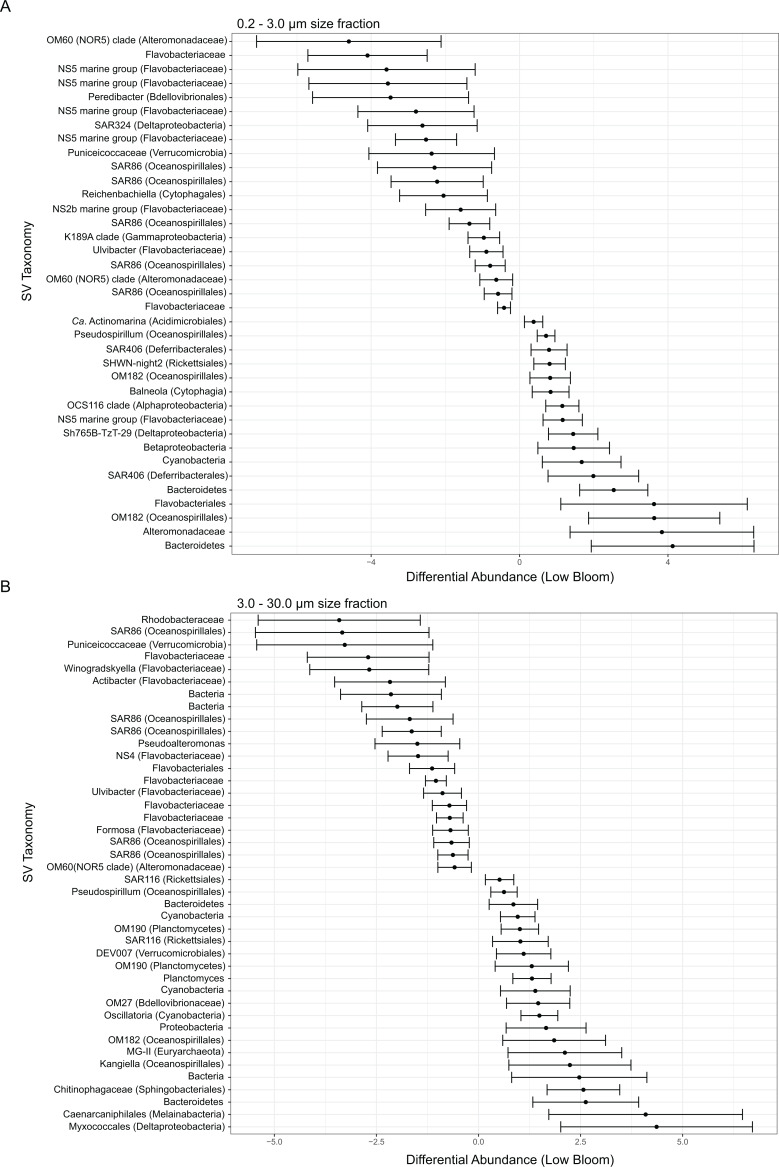
Microbial taxa enriched or depleted under low bloom conditions in (A) the smallest size fraction and (B) the intermediate size fraction. The frequencies of microbial taxa shift with bloom level. Each row represents an individual microbial sequence variant and the difference in its relative abundance under low bloom conditions compared to the overall mean (represented by *x* = 0). Points to the right of zero indicate enrichment under low bloom conditions; points to the left indicate depletion. Error bars display two standard deviations from the mean.

**Figure 4:**
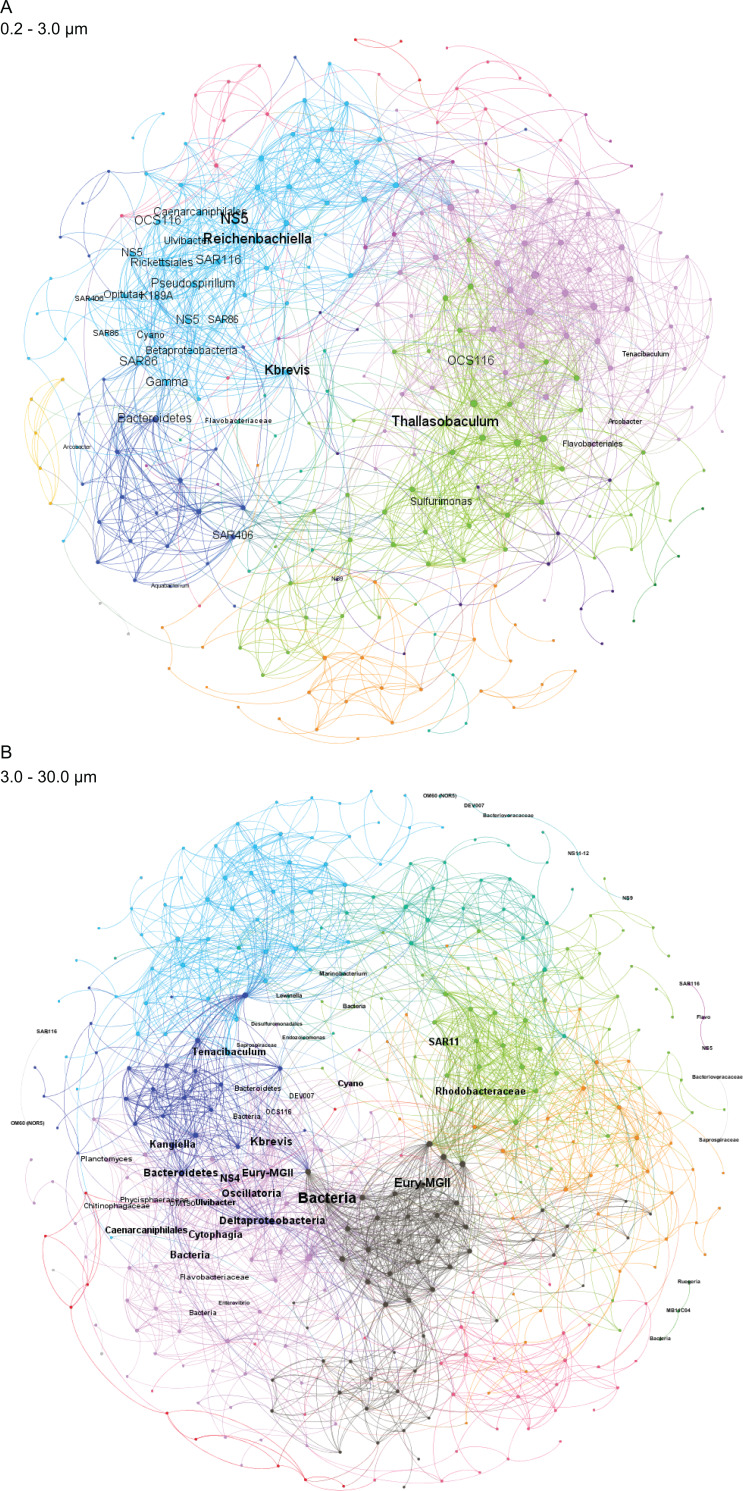
Microbial taxa and *K. brevis* are interconnected in both small and intermediate size fractions. Correlation networks for the small (A) and intermediate (B) size fractions show *K. brevis* is an ecological hub with high connectivity to several microbial groups, including SAR86, Reichenbachiella, and Thalassobaculum in the small size fraction and SAR11, Marine Group II euryarchaeota, and unidentified bacteria in the intermediate size fraction. Labeled nodes represent taxa significantly correlated with *K. brevis,* and the font size is proportional to the weighted degree of the node. In the intermediate size fraction, the nodes of five modules with no connections to the main network are also labeled.

To identify individual SVs contributing to these broad taxonomic patterns and statistically verify apparent differences, we applied two analyses, a binomial regression-based test (using corncob) to identify SVs significantly enriched/depleted in the high bloom dataset and a network analysis (using MINE) to identify SVs significantly correlated with *K. brevis* cell counts. For the small size fraction, corncob identified 20 SVs enriched and 17 SVs depleted under high bloom conditions (Fig. 3). For the intermediate size fraction, corncob identified 21 SVs enriched and 21 depleted under high bloom conditions (Fig. 3). MINE analysis further identified 31 and 34 SVs significantly associated with *K. brevis* in the small and intermediate size fraction networks, respectively (Fig. 4, Tables S5–S7). Network modularity was 0.537 and 0.605 for the small and intermediate size fraction datasets, respectively. Modularity is a value between -1 and 1 measuring the degree to which data features (nodes, in this case representing taxa) partition into modules (communities of connected nodes). These positive values indicate a greater number of connections between nodes within modules than expected by chance. The small and large size fraction networks contained 13 and 15 modules, respectively, with modules ranging in size from two to 56 (small fraction) or two to 69 (intermediate fraction) nodes. The largest module occurred in the intermediate size fraction and included the *K. brevis* node.

In the smallest size fraction, 25 SVs were both significantly associated with bloom state (either high or low; as identified by corncob) and significantly associated with *K. brevis* counts (MINE). For 11 of these SVs, the MIC value, a measure of two-variable dependence that is approximately equal to R^2^ but considers both linear and nonlinear associations, was greater than 0.99 (Table S8). These SVs included four flavobacteria, three Gammaproteobacteria, one member of the Alphaproteobacteria, two non-flavobacteria Bacteroidetes, and one member of the phylum Cyanobacteria.

In the intermediate size fraction, which presumably contained the bulk of *K. brevis* cells before filtration, 29 SVs were identified as associated with both bloom state (either high or low) and *K. brevis* counts; 10 of these had MIC values >0.99 (Table S8). Of these 29, 8 were enriched in high bloom conditions (Table 2). These included four flavobacteria, as well as SVs of the Puniceicoccaceae, SAR86, and Alteromonadaceae groups; SAR86 was also detected by both corncob and MINE analysis of the small size fraction. One of the 8 SVs could only be classified to the domain Bacteria. The remaining 21 SVs, which included three planctomycetes and a member of the MGII euryarchaeota, were enriched under low bloom conditions.

### Metabolomes

**Figure 5:**
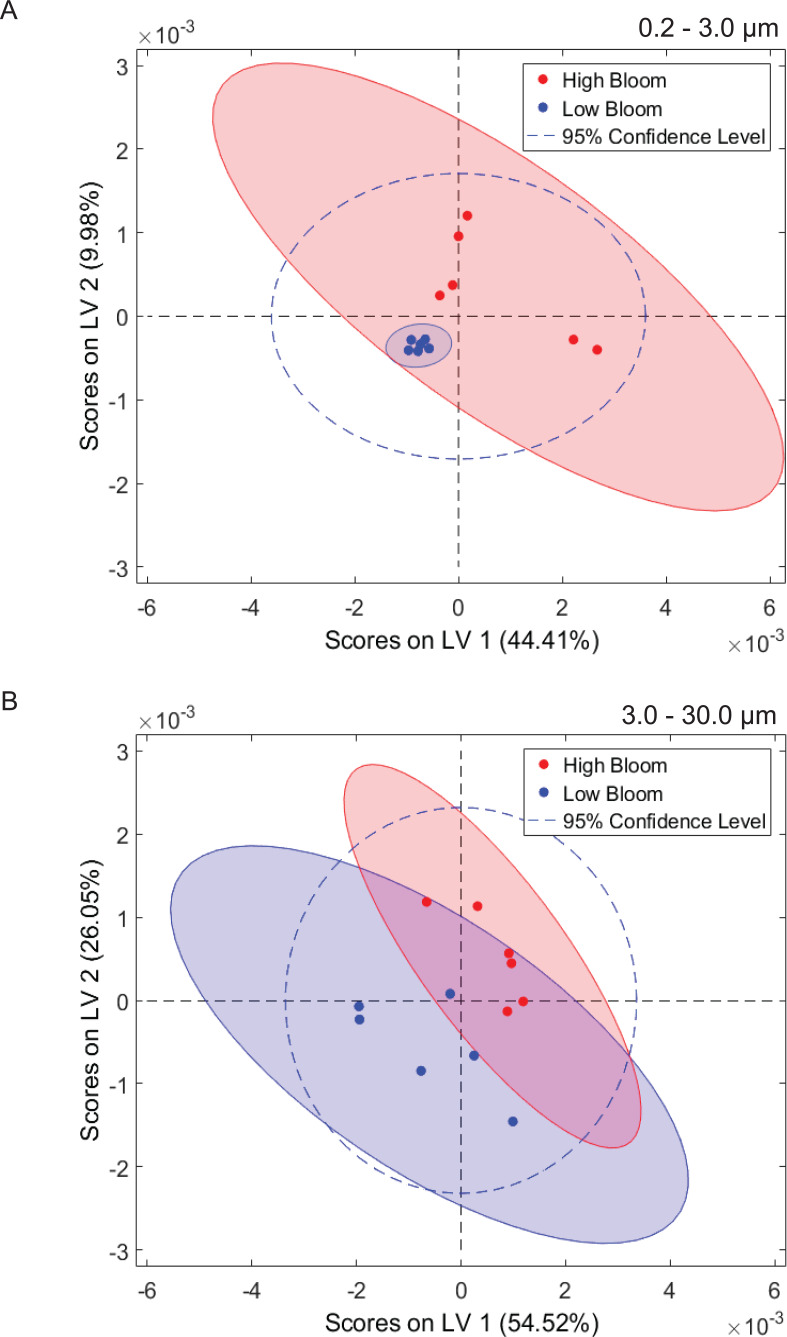
Phytoplankton and microbial community metabolomes differ based on bloom level. Partial least squares discriminate analysis (PLS-DA) for ^1^H NMR spectra differentiates lipid-soluble metabolomes of the microbial communities associated with high (<100 cells/mL of *K. brevis*; red) and low (<100 cells/mL of *K. brevis*; blue) bloom levels of *K. brevis*. (A) Metabolomes from the small size fraction primarily separate along the first latent variable (LV 1) and second latent variable (LV 2) with related NMR features accounting for 44% and 10% of the variance among samples, respectively. (B) Metabolomes from the intermediate size fraction primarily separate along the second latent variable (LV 2) and first latent variable (LV 1) with related NMR features accounting for 26% and 55% of the variance among samples, respectively. Tighter clustering among the high bloom samples indicates chemical profiles with greater similarity compared to those of the low bloom samples.

Multivariate statistical analysis (PLS-DA) of ^1^H NMR spectra representing size-fractionated plankton community metabolomes differentiated samples from high and low bloom sites (Fig. 5). High bloom metabolomes were less variable among samples compared to low bloom metabolomes for the intermediate size fraction, as shown by the tighter intra-class clustering and significantly smaller 95% confidence ellipse for samples in the high bloom class (Fig. 5B). Based on the non-overlapping confidence interval areas, the differentiation of high bloom and low bloom metabolomes was greatest in this intermediate size fraction containing *K. brevis*, other phytoplankton, and their attached bacteria. For the smallest size fraction containing free-living bacteria, metabolomes of the low bloom samples clustered tightly within the high bloom confidence interval (Fig. 5A). In contrast, the high bloom samples of this smallest size fraction showed greater variability. (Note that we did not generate metabolome data from extracts of the >30 µm fraction.)

**Figure 6:**
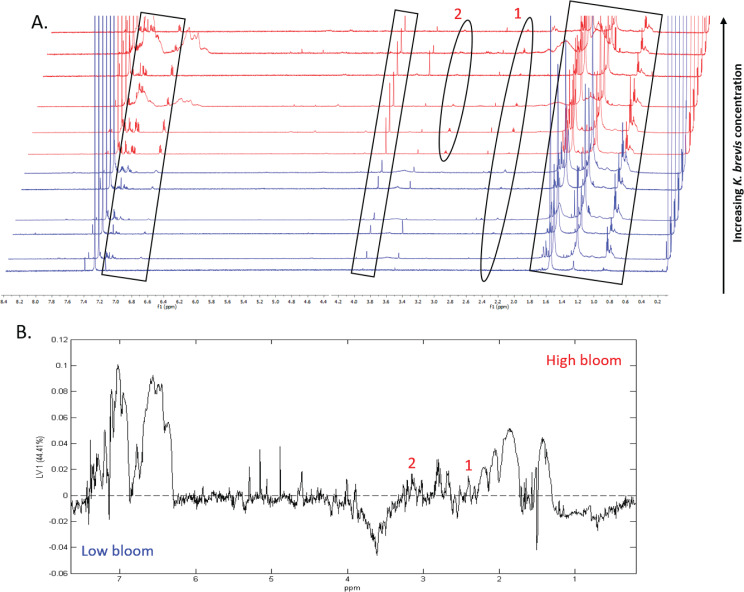
Specific metabolites associated with bacteria and small cells vary in presence and intensity in high and low bloom metabolome lipid-soluble extracts from the small size fraction. (A) Chemical profiles based on ^1^H NMR spectra from all 12 samples. Boxed peaks represent major filter contaminants and were disregarded during individual spectral analysis. High bloom (>100 cells/mL of *K. brevis*; spectra shown in red) samples were associated with more prominent signals at chemical shifts 2.35 ppm (“1”) compared to low bloom samples (<100 cells/mL of *K. brevis*; spectra shown in blue). Some but not all high bloom samples contained peaks at 3.15 ppm (“2”) that were absent in all low bloom samples. Overall, both high and low bloom individual profiles varied in peak presence/absence and high bloom profiles had more prominent peaks than low bloom samples. (B) PLS-DA latent variable 1 loadings plot of ^1^H NMR spectra accounting for 44% of the variance among bloom extract chemical profiles. Peaks along the positive y-axis primarily correspond to ^1^H-NMR spectral peaks from the high bloom extracts, and peaks along the negative y-axis primarily correspond to ^1^H-NMR spectral peaks from the low bloom extracts.

**Figure 7:**
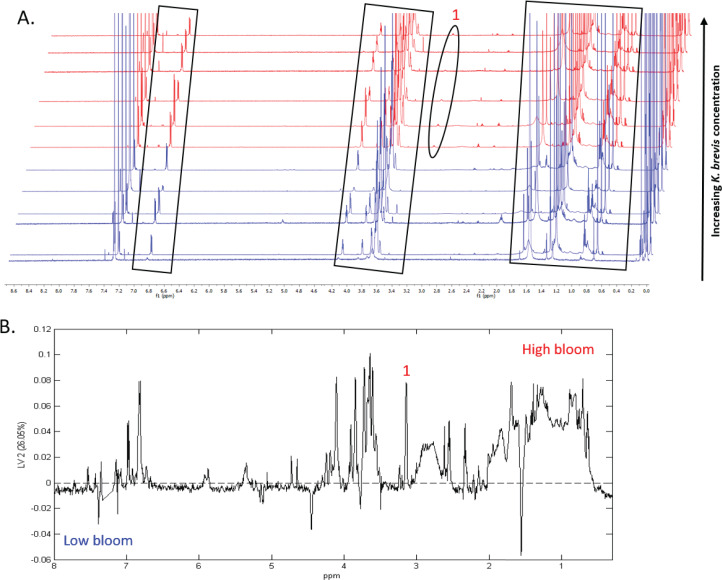
Specific metabolites associated with phytoplankton and large cells vary in presence and intensity in high and low bloom lipid-soluble metabolome extracts from the intermediate size fraction. (A) Chemical profiles based on ^1^H NMR spectra for all 12 samples. Boxed peaks represent major filter contaminants and were disregarded during individual spectra analysis. Most high bloom extracts share a chemical signature at 3.15 ppm (“1”) that is not found in the low bloom extracts. Otherwise, peak presence/absence and intensity did not consistently differ between the six high bloom (>100 cells/mL of *K. brevis*; spectra shown in red) and six low bloom (<100 cells/mL of *K. brevis*; spectra shown in blue) samples. Rather, variation was observed among individual profiles in both the high bloom and low bloom set. (B) PLS-DA latent variable 2 loadings plot of ^1^H NMR spectra accounting for 26% of the variance among chemical extracts. Peaks along the positive y-axis primarily correspond to 1H-NMR spectral peaks from the high bloom extracts, and peaks along the negative y-axis primarily correspond to 1H-NMR spectral peaks from the low bloom extracts.

^1^H NMR spectral analysis of lipid-soluble bloom extracts was consistent with the presence of aromatic compounds (with peaks between 6.8 ppm and 7.4 ppm) (Figs. 6A, 7A). These features were present in some high bloom extracts of the intermediate size fraction, but not in all high bloom samples, and in fewer low bloom samples (Fig. 7A). The enhanced concentrations of aromatic compounds in the high bloom extracts was made clear by signals at 6.8, 7.1, and 7.4 ppm in the loadings plot being positive on latent variable 2 (Fig. 7B) which best separated high and low bloom extracts in the scores plot (Fig. 5B). Similar NMR signals (6.8–7.4 ppm) in the smallest size fraction were also consistent with enrichment of aromatic compounds in high bloom samples with these indicative peaks positive on latent variable 1 (Fig. 6B), which best separated high and low bloom extracts in the scores plot (Fig. 5A), and largely only present in high but not low bloom extracts (Fig. 6A). Broad singlets, at 4.3 and 4.7 ppm, and peaks with varying multiplicities at 3.2–3.3 ppm, were indicative of alcohol, amine, ester, or halogen-containing metabolites, which occurred in the intermediate size fraction in both high and low bloom samples (Figs. 6A, 7A). These signals occurred more often and with greater prominence in the high bloom extracts, as shown by the positivity on latent variable 2 (Fig. 7B), although they were not found in all samples in either bloom level (Fig. 7A). Peaks in similar spectral regions (3.1–3.2 and 4.0–4.9 ppm) were found in the smallest size fraction (Fig. 6A); as in the intermediate size fraction, their positivity on latent variable 1 indicates these peaks drove the variation between the high and low bloom extracts primarily by their prominence in the high bloom extracts (Fig. 6B).

**Table 2:** SVs from the intermediate size fraction both significantly enriched in high bloom samples and significantly correlated with *K. brevis*, and the corresponding maximal information coefficients. Taxonomy is provided at the highest identified level.

**SV Taxonomy**	**MIC**
Bacteria; Bacteroidetes; Flavobacteriia; Flavobacteriales; Flavobacteriaceae; uncultured	0.99
Bacteria; Bacteroidetes; Flavobacteriia; Flavobacteriales; Flavobacteriaceae; Ulvibacter	0.99
Bacteria	0.99
Bacteria; Verrucomicrobia; Opitutae; Puniceicoccales; Puniceicoccaceae	0.74
Bacteria; Bacteroidetes; Flavobacteriia; Flavobacteriales; Flavobacteriaceae	0.74
Bacteria; Proteobacteria; Gammaproteobacteria; Oceanospirillales; SAR86 clade; uncultured bacterium	0.70
Bacteria; Proteobacteria; Gammaproteobacteria; Alteromonadales; Alteromonadaceae; OM60(NOR5) clade	0.70
Bacteria; Bacteroidetes; Flavobacteriia; Flavobacteriales; Flavobacteriaceae	0.70

Additional NMR signals were consistent with the presence of lipids in both size fractions. These features, which include those within the vinylic region (5.0–6.0 ppm), alkyl region (1.2–1.6 ppm), allylic region (1.8–1.9 ppm), and the region adjacent to carbonyls (2.1–2.9 ppm) (Figs. 6A, 7B), also drove the variation between high and low bloom metabolomes (Figs. 5A & 5B). The majority of statistically significant NMR signals separating the two bloom levels were attributed to the high bloom extracts, as shown by the abundance of signals in lipid-related regions that were positive on latent variables 1 (Fig. 6B) and 2 (Fig. 7B) in the loadings plots for the small and intermediate size fractions, respectively. While these features were not present in all samples, they occurred in more of the high than low bloom extracts for both size fractions. These patterns are represented in the ^1^H NMR spectra, where exemplary peaks that vary between high and low bloom metabolomes are numbered both in the raw spectra (Figs. 6A, 7A) and in the loadings plots (Figs. 6B, 7B). Signals indicative of brevetoxins were not detected in any of the spectra. Overall, statistical analyses indicate high bloom metabolomes were richer in lipids and aromatic metabolites compared to low bloom metabolomes (Figs. 6B, 7B), with substantial site-to-site variation in chemical profiles (Figs. 6B, 7B).

### Sparse canonical correlation analysis

**Figure 8:**
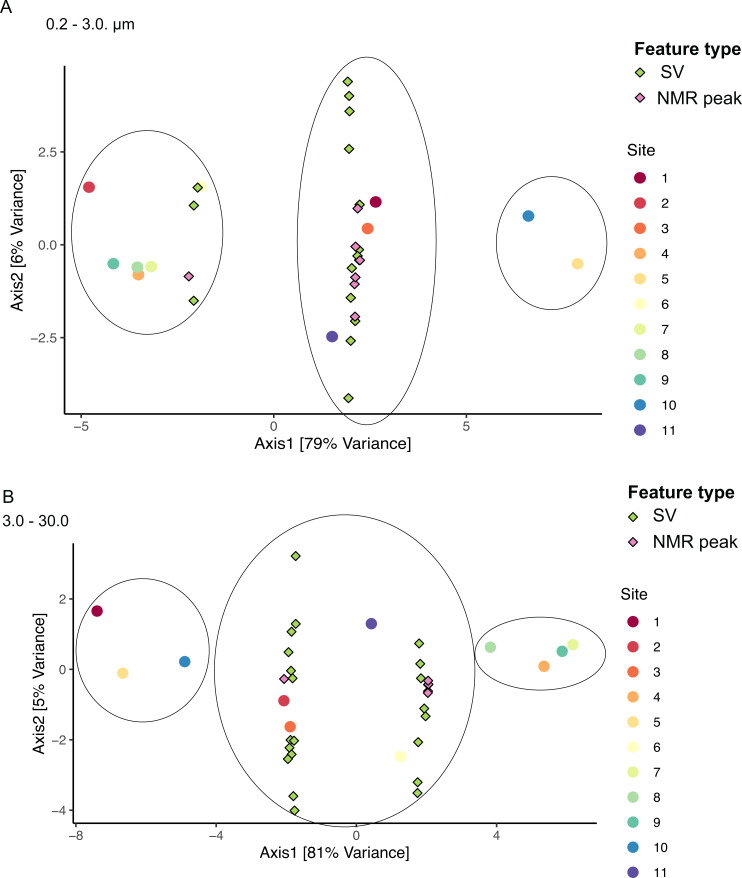
Sample groupings are driven by combinations of microbial taxa and chemical features. Sparse canonical correlation analysis (sCCA) of combined microbiome and metabolome data show sample groupings by microbial SVs and NMR spectral peaks. Principal Component Analysis triplots show highest separation along Axis 1 for (A) the small size fraction and (B) the intermediate size fraction. Sample groupings are circled for clarity.

We applied sCCA to identify features driving separation of combined microbiome and metabolome data. Samples of the smallest size fraction separated largely along one axis (79% of variation) into three main groups, as follows: (1) sites 2, 4, 6, 7, 8, 9, (2) sites 1, 3, 11, and (3) sites 5, 10 (Fig. 8A). Sixteen SVs and seven NMR peaks were identified as driving this separation (Table S9). Samples of the intermediate size fraction separated into two groups along one axis (94% of variation), as follows: (1) sites 1, 2, 3, 5, 10, 11, and (2) sites 4, 6, 7, 8, 9 (Fig. 8B). Six SVs and 52 NMR peaks were identified as driving this separation (Table S9).

## Discussion

Despite the ubiquity and increasing ecological impacts of HABs, the biological factors affecting bloom formation, intensity, and duration remain poorly understood. Bacteria and phytoplankton form intimate associations that include a range of positive, neutral, and negative interactions; it is therefore logical to hypothesize there exist features of microbial communities that correlate with blooms and affect bloom dynamics. Sampling over the natural spatial heterogeneity of coastal HABs is one important way of assessing bloom microbial and chemical features. However, the onset and magnitude of blooms are challenging to predict. Environmental data sets spanning gradients in bloom intensity, like the ones we present here, are therefore highly valuable.

In June 2018, a renewed boost of an ongoing *K. brevis* bloom resulted in high cell concentrations along the west coast of Florida (Fig. S1). While this bloom would ultimately turn into a severe, widespread, and long-lasting ecological phenomenon (Perkins, 2019), the early stages featured coastal areas differentially impacted by *K. brevis*. We sampled from sites of varying bloom levels in and around Sarasota Bay and Tampa Bay to assess microbiome and metabolome composition across a gradient of *K. brevis* cell concentration. To our knowledge, only one study has investigated natural microbial communities associated with different *K. brevis* cell concentrations (Jones et al., 2010). That study used 16S rRNA gene clone libraries to identify microbial taxonomic differences between “high” and “low” *K. brevis* levels. The samples were collected over several years, spanned a range of environmental variables, and were not size-fractionated, preventing differentiation of particle-associated from free-living microbial communities. In contrast, we sampled a *K. brevis* bloom in a relatively constrained geographic area over a 48-hour period to control for spatial and temporal variation. We also partitioned samples based on particle size to test for differences between free-living microbes (0.2–3.0 µm fraction) that may not be in direct contact with *K. brevis,* compared to microbial communities associated with larger size fractions (3.0–30 µm and >30 µm). The larger fractions presumably include the majority of phytoplankton cells (including *K. brevis*), as well as microbes directly attached to phytoplankton or zooplankton, or to other organic or inorganic particles.

Tampa Bay is the largest of the Florida estuaries, and water movement around the mouth of the bay is heavily influenced by tidal flow and river input (Weisberg & Zheng, 2006). Our sampling sites included sites directly exposed to the Gulf of Mexico as well as inner bay sites in Sarasota Bay and lower Tampa Bay (Fig. 1). Tidal patterns in this area are complex and replete with eddies, such that water around the mouth of Tampa Bay is well-mixed. However, salinity data from our sampling period show there was no major freshwater forcing in either the northern (low bloom) or southern (high bloom) sampling site groups (http://ocgweb.marine.usf.edu/Models/FVCOM/month_tb_ch.php?year1=2018&mon1=jun). Our sites are therefore likely relatively similar to each other in physicochemical parameters and varied mainly in the level of *K. brevis* at the time of sampling. The exception is site 12, which was located at a marina situated within an intracoastal waterway and featured minimal mixing and flow relative to other sites. Furthermore, water quality at site 12 was visibly lower than at all other sites, with hydrocarbon slicks at the surface and prevalent debris from boats and human activity. Microscopy counts confirmed this site as an outlier in phytoplankton community composition (Table 1). Microbiome sequencing of the intermediate size fraction from site 12 resulted in too few reads for meaningful analysis (Table S1), and the largest size fraction differed notably in composition from that of other sites (Fig. S2). However, the small size fraction sequenced well and did not skew patterns of alpha or beta diversity, so we included it in molecular and metabolomics statistical analyses.

Surprisingly, we did not detect *K. brevis* or other dinoflagellates in the taxonomic analysis of chloroplast 16S rRNA gene sequences. The primers used in our analysis are designed for bacterial and archaeal 16S rRNA genes and are not assumed to universally capture the chloroplast 16S rRNA gene pool. Indeed, the six *K. brevis* sequences in the PhytoRef plastid database differ from our reverse primer (806R) by a one-nucleotide mismatch, which may have hindered amplification. Moreover, *K. brevis* cells are highly fragile and may have lysed during filtration, with dissolved cell contents and small particulates passing through the filters. Rather, our chloroplast sequence analysis identified several species of diatoms, along with other algal groups like prymnesiophytes and chlorophytes (Table S2). These results partly corroborate the cell count data and provide additional qualitative description of the sites, but should not be interpreted as a quantitative measurement of phytoplankton composition (Table S2).

Among the factors considered in our analysis, size fraction most strongly affected microbiome composition and alpha diversity (Fig. 2 (*p* = 0.001), Figs. S2 , S3). This finding is supported by extensive literature on differentiation of free-living and particle-associated microbial communities (Hollibaugh, Wong & Murrell, 2000; Grossart et al., 2005; Eloe et al., 2011; Ganesh et al., 2014). As in previous studies, we found members of the phyla Planctomycetes, Verrucomicrobia, and Bacteroidetes increased in frequency with size fraction (Fig. S4). These taxa can metabolize algal-derived organic matter (Teeling et al., 2012; Kirchman, 2002) and are likely important components of communities attached to particles. In the largest size fraction, these particles may include zooplankton or even microplastics. In contrast, the smallest size fraction (0.2–3.0 µm) presumably contained most of the free-living bacteria and archaea, and was enriched in all Proteobacterial classes except for Deltaproteobacteria (Fig. S4). We compared our results with a study on size-fractionated microbiomes associated with blooms of two non-*K. brevis* dinoflagellate species in Northport Bay, New York (Hattenrath-Lehmann & Gobler, 2017). In that study, one of the most commonly observed flavobacteria in the small (0.2–20 µm) size fraction belonged to the uncultured NS5 lineage. This taxon was significantly associated with *K. brevis* in the smallest size fraction of our data, suggesting it may be a generalist competitor enriched during dinoflagellate blooms. In contrast, unlike our results, the New York study found lower levels of Bacteroidetes and higher levels of Proteobacteria in the large (>20 µm) size fraction relative to the small size fraction during bloom peaks. Dinoflagellate microbiomes may therefore vary by species and geography, despite similarities in factors like particle composition (i.e., dinoflagellate cell wall structure) and secreted nutrients.

Microbiome composition also varied with bloom level. The separation of taxonomic profiles by bloom level, while not as strong as that among size fractions, was significant along the second principal component (Fig. 2; PERMANOVA *p* = 0.002). This suggests that particle-associated microbes, including those attached to larger (>30 µm) as well as smaller (3–30 µm) particles, change with *K. brevis* cell concentrations*,* as do free-living microbes. There are several potential explanations for these differences. Communities may shift because of bloom-driven differences in nutrient availability as primary production increases. Phytoplankton release photosynthates into the water column in the form of dissolved organic matter, thereby altering local organic carbon levels and providing a nutrient boost to bacterioplankton (Berman & Holm-Hansen, 1974; Smith & Wiebe, 1976; Baines & Pace, 1991). Furthermore, *K. brevis* can incorporate a wide range of organic nutrients, including ammonia, nitrate, urea, humic acids, and amino acids (Killberg-Thoreson et al., 2014) and organic matter derived from other extracellular exudates of other phytoplankton species (Sipler et al., 2013). *K. brevis* may therefore directly compete with bacteria for these and other nutrients. Bloom progression can result in a succession of microbial community composition as taxa specialized for different nutrient or carbon regimes increase or decrease in abundance (Joint et al., 2002; Teeling et al., 2012). As such, we may have captured only two of potentially many microbial community states associated with a *K. brevis* HAB.

The pool of bioactive metabolites accompanying a bloom may also influence the presence and abundance of surrounding microbes. *K. brevis* produces a wide array of compounds in addition to brevetoxins; indeed, many metabolites remain unidentified including those responsible for most of the allelopathy against other phytoplankton species (Poulson-Ellestad et al., 2014; Poulin et al., 2018b). Bioactive metabolites may inhibit microbes either directly (through antimicrobial activity) or indirectly (by changing the plankton community structure and nutrient availability). Previous work showed that brevetoxins affect both bacterial cell abundance and community composition, although most of the effects were observed in microbial assemblages from sites not frequently exposed to *K. brevis* blooms (Sipler et al., 2014), which is not the case for surface waters around Sarasota Bay. In the current study, brevetoxins were not identified in our samples, so we were unable to determine potential brevetoxin-microbe linkages. However, brevetoxins were likely present but went undetected due to the lysis of fragile *K. brevis* cells during filtration. Extracellular brevetoxins from lysed cells are a common feature of *K. brevis* blooms (Steidinger & Baden, 1984; Pierce et al., 2003). It is therefore reasonable to assume that brevetoxins were present at sites with *K. brevis* and may have had some unresolvable impact. We did not measure whether bacterial cell abundances were affected by *K. brevis* bloom levels; however, the observed taxonomic differences between bloom levels suggest some bacteria may be inhibited by one of the aforementioned mechanisms. For most taxa, the pattern of enrichment based on bloom condition varied depending on the size fraction being examined (Fig. S5). Toxin production for the purpose of inhibiting bacterial growth via cell lysis or disrupted metabolism may be a strategy to eliminate potential competitors as a bloom progresses. Some microbes may themselves be induced to produce chemical defenses to avoid being grazed; these compounds could also be reflected in the planktonic metabolome and their presence may induce antimicrobial metabolite production in *K. brevis*.

Microbes attached to *K. brevis* or other phytoplankton may interact with algal cells through diverse mechanisms, potentially including direct interaction with secreted metabolites. Taxa found in the intermediate (3–30 µm) size fraction include those most likely to engage in direct-contact relationships with phytoplankton cells. Eight SVs from the intermediate size fraction were identified as being both enriched in high-bloom conditions and associated with *K. brevis* cell counts (Figs. 3, 4, Table 2). These included four SVs of the flavobacteria, a group known to comprise major components of the microbial community associated with phytoplankton blooms (Kirchman, 2002; Pinhassi et al., 2004; Grossart et al., 2005; Teeling et al., 2012). Although these SVs have yet to be characterized, we hypothesize they are part of a *K. brevis*-associated microbiome, potentially one with novel taxa. Indeed, one of the eight SVs could only be classified to the domain Bacteria (Table 2). It is possible this SV represents a group that, by virtue of association with a specific host and the challenge of cultivating host-associated microbes, is underrepresented in sequence databases and culture collections, but plays an important role in *K. brevis* physiology and ecology. In the small size fraction, along with flavobacteria and SAR86 SVs, representatives of the deltaproteobacterial clade SAR324 and the predatory bacterial family Bacteriovoracaceae varied significantly based on bloom condition (Table S8). Recovery of these groups in the small size fraction implies a lack of direct physical attachment to larger cells or particles at the time of sampling. These taxa instead may have been responding to higher levels of dissolved organic matter or chemical cues produced by *K. brevis*.

Correlation networks showed that *K. brevis* is strongly associated with co-occurring microbial taxa (Fig. 4). Indeed, *K. brevis* may be a “hub” within the community, as it had many connections within its own module, as well as connections across modules. Surprisingly, the *K. brevis* node had higher connectivity (more and stronger connections) in the small size fraction network compared to that of the particle-associated fraction. These results may indicate it has an attached microbiome that is flexible in composition and varies among or within *K. brevis* populations, while the smaller number of taxa strongly linked to *K. brevis* may represent a “core” attached microbiome. Conversely, the effects of high *K. brevis* levels on the free-living microbial community may consistently affect a wider range of taxa. This hypothesis is supported by the sparse CCA results, which identified many more SVs driving sample separation in the small size fraction compared to the intermediate fraction (16 vs. 6). Given the interconnected ecological webs that characterize phytoplankton blooms, and microbial communities in general, the complexity of these networks is not surprising. Combined with the results from the binomial regression model, these networks provide a baseline for assessing HAB microbial communities at various stages of bloom intensity. The network results also identify specific microbes (those with the strongest connections to *K. brevis*) for study as HAB-associated taxa that may play important roles in the intensity and duration of *K. brevis* blooms, potentially via chemical interdependencies similar to those common in other phytoplankton-bacteria interactions (Seyedsayamdost et al., 2011; Amin et al., 2015; Seymour et al., 2017; Cirri & Pohnert, 2019).

The observed differences in microbial communities between high and low bloom levels are coincident with differences in the associated metabolomes. This observation is expected given the potential for HABs to affect the metabolism of co-occurring organisms by producing bioactive molecules or altering nutrient and carbon availability. These effects can be elucidated in part by metabolomics. The best-studied *K. brevis* metabolites, brevetoxins, did not emerge as the chemicals differentiating high bloom from low bloom sites, and in fact were not detected in the metabolomic analysis, likely because *K. brevis* compounds were lost due to cell lysis. In addition to brevetoxins, *K. brevis* produces a suite of allelopathic compounds (Prince et al., 2010; Poulin et al., 2018b) which have recently been linked to metabolic impacts on phytoplankton competitors (Poulson-Ellestad et al., 2014; Poulin et al., 2018a). This allelopathy and resulting changes in phytoplankton community structure could be partially responsible for the discrete clustering of high versus low bloom lipid-soluble metabolomes in both the small and intermediate size fractions (Fig. 5). Other harmful algal bloom-producing dinoflagellates, including *Alexandrium* spp., are allelopathic towards phytoplankton competitors (Fistarol et al., 2004) and produce compounds that impact the composition and abundance of microbial communities (Weissbach, Tillmann & Legrand, 2010). It is therefore reasonable to hypothesize that *K. brevis’* metabolites exert similar ecological effects, serving as chemical drivers that result in microbiome and phytoplankton community composition changes during a HAB.

The impact of *K. brevis* allelopathy on metabolomes of co-occurring phytoplankton and microbes may be represented in the inter-sample metabolome variation, which differed based on size fraction. High bloom metabolomes in the intermediate size fraction were less variable than both low bloom metabolomes of the same size fraction and high bloom metabolomes from the smallest fraction. The greater similarity among metabolomes from high versus low bloom sites may have resulted from *K. brevis* allelopathic effects on the phytoplankton community (characterized by solitary diatoms, colonial diatoms, and other dinoflagellates) under high bloom conditions (Fig. 5B, Table 1), as discussed above. In contrast, metabolomes from the smallest size fraction were more variable (Fig. 5A), whereas the low bloom metabolomes clustered most tightly among all of the subsets, suggesting that *K. brevis* concentration may diversify the ecological interactions and subsequently the metabolite profiles associated with free-living microbial communities at the bloom concentrations we measured.

The differential clustering of high versus low bloom metabolomes for both size fractions was supported by differential enrichment of lipids and aromatic compounds. Overall, high bloom metabolomes had relatively higher concentrations of both aromatic and lipid metabolites (Figs. 6, 7). The suite of allelopathic compounds produced by *K. brevis* is known to include compounds with aromatic functional groups (Prince et al., 2010). Other bacterial groups are known to produce aromatic oxygen- and nitrogen-containing compounds and lipids in response to HAB dinoflagellates (Amaro et al., 2005; Wang et al., 2005; An et al., 2015); some of these bacteria, including flavobacteria, may have algicidal effects on *K. brevis* (Roth, Mikulski & Doucette, 2008; Roth, Mikulski & Doucette, 2008). Thus, it is possible that the high bloom metabolomes captured a greater number of antagonistic bacteria-phytoplankton interactions, and the resulting shifts in phytoplankton and bacterial intracellular metabolomes, compared to low bloom metabolomes. However, algicidal and allelopathic interactions are likely two of many non-exclusive mechanisms contributing to differential metabolome structuring under high versus low bloom conditions. Other possibilities include, but are not limited to, antimicrobial metabolites produced by *K. brevis* or other phytoplankton species and metabolites facilitating mutualistic interactions between phytoplankton and bacteria. Although most studies identifying specific bacteria-algae-chemical interactions have been conducted in lab settings, similar and perhaps more complex interactions likely occur during a natural bloom and may account for the metabolome variation we observed.

When microbiome and metabolome data were combined in the sCCA, samples did not separate exclusively by bloom level (Fig. 8). This is likely due to the relatively high among-sample variability within low and high bloom sample groups when both biological and chemical data are considered. Unfortunately, the chemical features identified by the sCCA largely consisted of artifacts of the NMR processing, so we could not derive any specific metabolite information from those results. However, several of the SVs that drove sample clustering in the sCCA were also identified by the regression and network analyses as being associated with *K. brevis* (Table S9). Moreover, both size fractions showed three clusters with similar sample composition, suggesting there are larger biological and chemical patterns that affect both free-living and particle-associated microbiomes and metabolomes. These consistencies point to underlying biological features that could emerge as strong drivers of bloom dynamics with more extensive sampling and higher replication.

## Conclusions

This study captures microbial and chemical diversity and composition patterns associated with the beginnings of a toxic algal bloom. We leveraged samples representing differences in *K. brevis* cell abundances to show that bloom level has a significant influence on both microbial taxonomic and metabolome structuring. This influence persists but is variable across biomass size fractions, reflecting differences in microbial lifestyle (free-living versus particle-associated). Furthermore, we identified eight bacterial sequence variants potentially associated with *K. brevis*. A logical next step is to quantify temporal patterns in microbes and metabolites associated with the beginning, middle, and end of a bloom. The dynamic and unpredictable nature of these blooms is an important challenge, but scientific and public interest in the causes and effects of *Karenia brevis* red tides provide ample motivation to plan appropriate sampling schemes and build off our findings of *K. brevis*-microbial connectivity.

##  Supplemental Information

10.7717/peerj.9493/supp-1 Supplemental Information 1 Cell counts of *Karenia brevis* in the spring and early summer 2018 *Karenia brevis* cell counts reported by the Florida Fish and Wildlife Conservation Commission show the harmful algal bloom had declined in the spring of 2018 before increasing in June, when we conducted our sampling. Data sources: Scripps Institution of Oceanography, National Oceanic and Atmospheric Administration, U.S. Navy, National Geospatial-Intelligence Agency, GEBCO Image Landsat/Copernicus, Google Earth.

10.7717/peerj.9493/supp-2 Supplemental Information 2 Samples within each size fraction had similar microbiome compositions Samples grouped by microbiome community composition. Each bar represents one sample composed of taxa resolved at the subphylum or class level. All taxa are in the domain Bacteria.

10.7717/peerj.9493/supp-3 Supplemental Information 3 Alpha diversity patterns differ by size fraction and bloom level Rarefaction curves of Faith’s phylogenetic diversity as a function of sequencing depth show diversity decreases with size fraction. Within each size fraction, alpha diversity metrics (Faith’s phylogenetic diversity and Pielou’s evenness) are not significantly different between high and low bloom samples (all *t*-test *p*-values > 0.05).

10.7717/peerj.9493/supp-4 Supplemental Information 4 Several groups of bacteria and archaea showed differential frequency patterns among the three size fractions (A) Taxa with a relative abundance >5% in at least one size fraction. (B) Taxa with relative frequencies <3%. Acidi = Acidimicrobiia (phylum Actinobacteria), Flavo = Flavobacteriia (phylum Bacteroidetes), Sphingo = Sphingobacteria (phylum Bacteroidetes), Cyano = Cyanobacteria (phylum Cyanobacteria), Alpha = Alphaproteobacteria, Delta = Deltaproteobacteria, Gamma = Gammaproteobacteria, IncSed = Proteobacteria Incertae Sedis, Verruco = Verrucomicrobia, Bacteria = unknown bacterial phylum, MGII = Marine Group II Euryarchaeota, Unid Cyano = unclassified Cyanobacterial subphylum, Deferri = Deferribacteres (phylum Deferribacteres), OM190 = OM190 (phylum Planctomycetes), Phycisph = Phycisphaerae (phylum Planctomycetes),Opitu = Opitutae (phylum Verrucomicrobia), Cytoph = Cytophagia (phylum Bacteroidetes), Beta = Betaproteobacteria. Boxes show the quartiles of frequency across samples and whiskers extend to show the rest of the distribution, except for outliers represented by diamonds. Lines within the boxes represent the median frequency value.

10.7717/peerj.9493/supp-5 Supplemental Information 5 Most subphyla and classes of bacteria and archaea had similar frequencies in the high (>100 cells/mL) and low (<100 cells/mL) *K. brevis* bloom sites (A) Average frequencies of taxa from the small size fraction. (B) Flavobacteriia, Deferribacteres, and Deltaproteobacteria in the intermediate size fraction were differentially abundant. Taxon names are abbreviated as in Fig. S3.

10.7717/peerj.9493/supp-6 Supplemental Information 6 Tables S1-S9

10.7717/peerj.9493/supp-7 Supplemental Information 7 R code for generating sparse CCA from microbiome and metabolome data

## Additional Information and Declarations

### DNA Deposition

The following information was supplied regarding the deposition of DNA sequences: Raw sequences are available in the NCBI Sequence Read Archive (BioProject): PRJNA530696.

### Data Availability

The following information was supplied regarding data availability: Sequence read counts, including chloroplast reads, and statistical test results are available in Tables S1–S9. The R code for the sparseCCA is available in a Supplemental File.
