# Supplementary material for: Microbial and chemical dynamics of a toxic dinoflagellate bloom
Source: PeerJ. 2020 Jul 21;8:e9493. doi: 10.7717/peerj.9493 (PMC7676380; doi:10.7717/peerj.9493)
Supplement: Supplemental Information 1 — Karenia brevis cell counts reported by the Florida Fish and Wildlife Conservation Commission show the harmful algal bloom had declined in the spring of 2018 before increasing in June, when we conducted our sampling. Data sources: Scripps Institution of Oceanography, National Oceanic and Atmospheric Administration, U.S. Navy, National Geospatial-Intelligence Agency, GEBCO Image Landsat/Copernicus, Google Earth. [file peerj-08-9493-s001.pdf]

A. B. C.

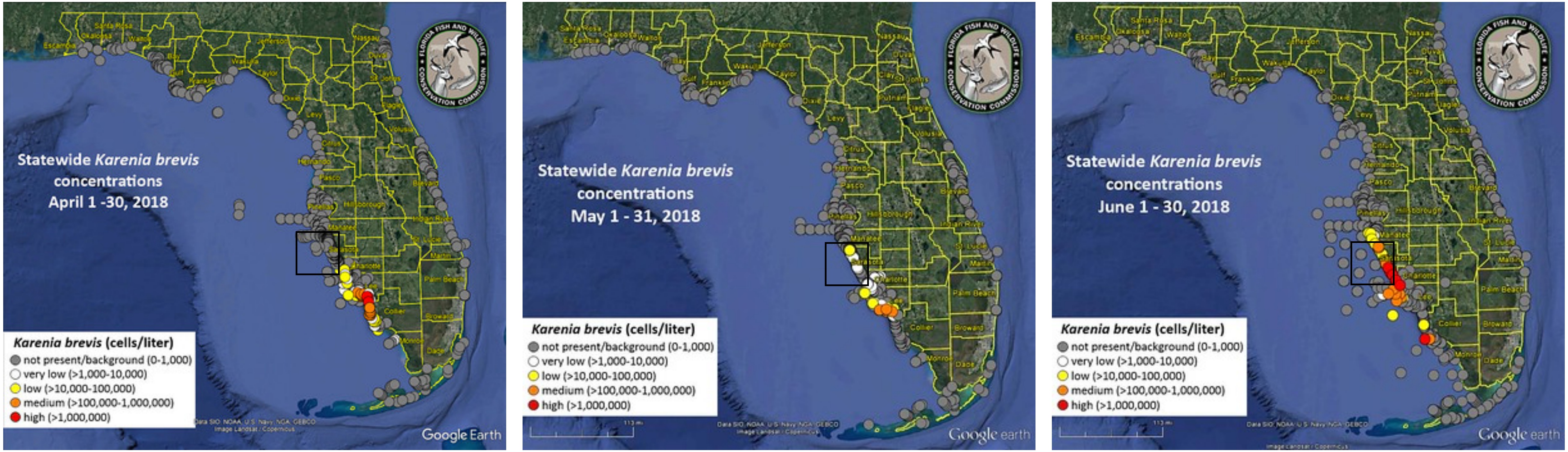

Supplementary Figure 1. *Karenia brevis* cell counts during April (A), May (B), and June (C) 2018 show June represented a renewed surge in bloom conditions in the sampling area (black box). Maps were provided by the Florida Fish and Wildlife Conservation Commission (FWWC) archive of HAB monitoring data (https://www.flickr.com/photos/myfwc/sets/72157635398013168/with/42439280324/). Data sources: Scripps Institution of Oceanography, National Oceanic and Atmospheric Administration, U.S. Navy, National Geospatial-Intelligence Agency, GEBCO Image Landsat/Copernicus, Google Earth.
